# Autologous menisci–cruciate ligament composite as a flap for soft tissue reconstruction following malignant bone tumor resection around the knee

**DOI:** 10.1002/cam4.5591

**Published:** 2023-01-09

**Authors:** Yu Xu, Yan Li, Yiwei Fu, Bo Wang, Tiao Lin, Changye Zou, Gang Huang, Jingnan Shen, Junqiang Yin

**Affiliations:** ^1^ Department of Orthopedic Oncology The First Affiliated Hospital of Sun Yat‐Sen University Guangzhou Guangdong China; ^2^ Guangdong Provincial Key Laboratory of Orthopedics and Traumatology Guangzhou Guangdong China

**Keywords:** bone neoplasms, limb salvage, reconstruction, soft tissue defect

## Abstract

**Background:**

Despite significant improvements in oncological treatment, the management of soft tissue defects following malignant tumor resection remains challenging. We investigated whether autologous menisci and cruciate ligament, which are traditionally discarded, can be recycled as a supplemental flap in repairing soft tissue defects following malignant bone tumor resection and endoprosthetic reconstruction around the knee.

**Methods:**

Four knee specimens were dissected to provide a basis for the design of the menisci–cruciate ligament composite. Then, 40 patients with bone malignancies around the knee were enrolled and underwent reconstruction with free or vascularized composite following malignant tumor resection. The clinical, radiographic, and functional outcomes of this technique were evaluated in >1‐year follow‐up in each patient and compared with 87 patients who suffered from bone malignancies around the knee and were treated by limb salvage but without composite at our center over the same period. During the follow‐up, a composite from one patient who underwent secondary amputation was retrieved and examined for in vivo remodeling.

**Results:**

Fourteen patients were treated with vascularized composite transfer (10 distal femurs and 4 proximal tibias) and 26 patients with free composite transfer (19 distal femurs and 7 proximal tibias). The composite can be used to cover the area of soft tissue defect from 22 to 48.38 cm^2^ (34.67 ± 6.48 cm^2^). With contrast‐enhanced ultrasound, peripheral rim healing and dotted blood flow signal at the side of anastomosis were detected on a patient 16 months after free composite transfer. Gross macroscopic remodeling and histopathologic analysis of a retrieved composite also indicated good healing with surrounding tissues and living cells in the composite. The complications and oncologic outcomes were comparable between study and control cohorts, but better Musculoskeletal Tumor Society (MSTS) score for patients reconstructed with composite (26.68 vs. 25.66, *p*  = 0.004). Of note, MSTS score was higher for patients reconstructed with composite at distal femur subdivision compared with the same subdivision in the control cohort (26.97 vs. 25.90, *p*  = 0.009). No statically significant difference was noted in complications, oncologic, and functional outcomes for patients reconstructed with free or vascularized composite.

**Conclusion:**

Autogenous menisci–cruciate ligament composite is an alternative option for soft tissue reconstruction. Either vascularized or free composite can be applied, depending on the size and localization of the defect.

## Graphical abstract

Autologous menisci and cruciate ligament can be recycled as a flap in repairing soft tissue defects following malignant bone tumor resection and endoprosthetic reconstruction around the knee.The recycled composite is safe and provides better Musculoskeletal Tumor Society (MSTS) score.

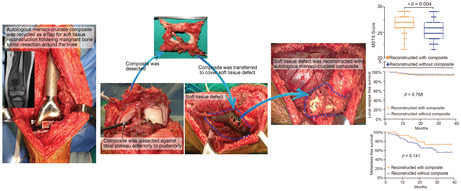

## 1 INTRODUCTION

Primary malignant bone tumors frequently occur at the metaphysis around the knee. Coupled with multidisciplinary approaches, limb salvage is now performed for nearly 90% of appendicular malignant bone tumors.^1^ Complete surgical resection with negative margins is the gold standard for both local control and overall survival.^2^ However, obtaining negative surgical margins may require extensive resection, causing prominent soft tissue defects and functional deficits.^3^ Although muscle or fasciocutaneous flaps generally provide favorable coverage, sometimes they do not, and then supplementary soft tissue is required to obtain adequate coverage.^4^, ^5^ In addition, while soft tissue coverage is the main goal in tumor cases, functional reconstructions are gaining popularity.^6^

For the knee joint, the epiphyseal plate and articular cartilage are barriers to tumor invasion in the meniscus and cruciate ligaments. We noticed that the menisci and cruciate ligaments were resected and discarded followed by endoprosthetic reconstruction. Inspired by others who used soft tissue flaps with no tumor invasion from the amputated limb to cover soft tissue defects,^7^, ^8^ we wondered whether menisci and cruciate ligaments, otherwise useless, could be recycled as a supplemental flap and coupled with other well‐described muscle flaps to ensure adequate soft tissue coverage.

Here, we first carried out a cadaver study to provide an anatomic basis for the surgical procedures. Then, we treated 40 patients with free or vascularized autogenous menisci–cruciate ligament composite to reconstruct soft tissue defects following malignant tumor resection and evaluated the effectiveness of the technique.

## 2 METHODS AND MATERIALS

This study was approved by the ethics committee (Application ID: [2021]119) and registered at ChiCTR (ChiCTR2100052249). All operations were performed per the Helsinki declaration, and informed consent was obtained from all patients.

### 2.1 Anatomic study

Four unpaired fresh‐frozen knees (two left and two right knees) without any scars from donated cadavers were involved in the study. The specimens were injected with 60% liquid latex mixed with red pigment and then fixed in 10% formaldehyde for 1 week. Subsequently, they were dissected.

### 2.2 Patients

Between December 2018 and December 2020, 40 patients (25 males and 15 females) afflicted with bone malignancies around the knee were prospectively enrolled as the study cohort. The inclusion criteria were as follows: (1) bone malignancy around the knee; (2) the knee joint cavity was confirmed to be free from tumor invasion; (3) without metastasis (no skip metastasis or distant metastasis); (4) no cancer embolus in blood, with preoperative contrast‐enhanced magnetic resonance imaging (MRI) and computed tomography. The exclusion criteria were as follows: (1) extension of the tumor into the knee joint, (2) intraarticular soft tissue malignant tumors, (3) suspected metastasis, and (4) pathologic fracture or preoperative operations contaminating the joint space.

The tumors were located in the distal femur (*n* = 29) and proximal tibia (*n* = 11). The mean age at the time of surgery was 16.3 years (range 7–45 years). The characteristics of the individuals are shown in Table S1. All patients underwent biopsies and pathology reviews before surgery. The preoperative evaluation involved plain radiography, computed tomography, and contrast‐enhanced MRI to define the extent of the tumor and initial staging. The standard treatment for these patients included neoadjuvant chemotherapy, surgery, and adjuvant chemotherapy.^9^

In parallel, a retrospective review of 106 patients who suffered bone malignancy around the knee and were treated by limb salvage but without composite between November 2018 and December 2020 at our institution was performed. Medical records were reviewed for patient demographics, tumor characteristics, performed treatments, operation records, and postoperative outcomes. Only patients with complete medical records and those with >12 months of clinical follow‐up were involved. Finally, a total of 86 patients (52 males and 34 females) were eligible as the control cohort, with a mean follow‐up period of 21 months (from 12 to 39 months). Patients were divided into two subdivisions, depending on the tumor location, one group consisted of patients with distal femur lesions (*n* = 58) and the other consisted of patients with the proximal tibial lesion (*n* = 28). The characteristics of the individuals are shown in Table S2.

### 2.3 Follow‐up and outcome assessment

Clinical and radiographic examinations were performed every 3 months for the first 2 years, every 4 months for 3–5 years, and annually thereafter. During clinical examination, extra attention was paid to patients' wound status and blood supply of composite by Doppler ultrasound. The diagnosis of prosthetic joint infection (PJI) was based on the Musculoskeletal Infection Society's definition.^10^ Functional outcomes were assessed with a Musculoskeletal Tumor Society (MSTS) score.^11^ Scores were calculated at the orthopedic oncology clinic 1 year postoperatively because most patients have completed both chemotherapy and rehabilitation by 1 year and the functional condition was stable.

### 2.4 Statistical analysis

Categorical variables were presented as numbers and percentages and continuous variables as median values. The chi‐square test was employed to compare the baseline characteristics between the study and control cohort, and the same subdivision, such as patients reconstructed with composite at the distal femur in the study cohort versus the one reconstructed at the distal femur but without composite in the control cohort. Local relapse‐free survival (LRFS) was defined as the time interval from the date of diagnosis to the date of local relapse or last follow‐up. Metastasis‐free survival (MFS) was defined as the time interval from the date of diagnosis to the date of metastasis or the last follow‐up. Kaplan–Meier curves were used to depict the oncologic outcomes including LRFS and MFS between the study and control cohort and the same subdivision. Then, log‐rank tests were employed to examine differences between survival curves. The MSTS score was compared between the study and control cohort, and the same subdivision using not unpaired *t*‐test. A two‐sided value of *p* < 0.05 was considered to indicate statistical significance. Statistical analysis was performed with SPSS version 22.0 software (IBM Corp.) and GraphPad Prim 8 (GraphPad Software Inc.).

## 3 RESULTS

### 3.1 Implications for design of composite based on anatomic study

**FIGURE 1:**
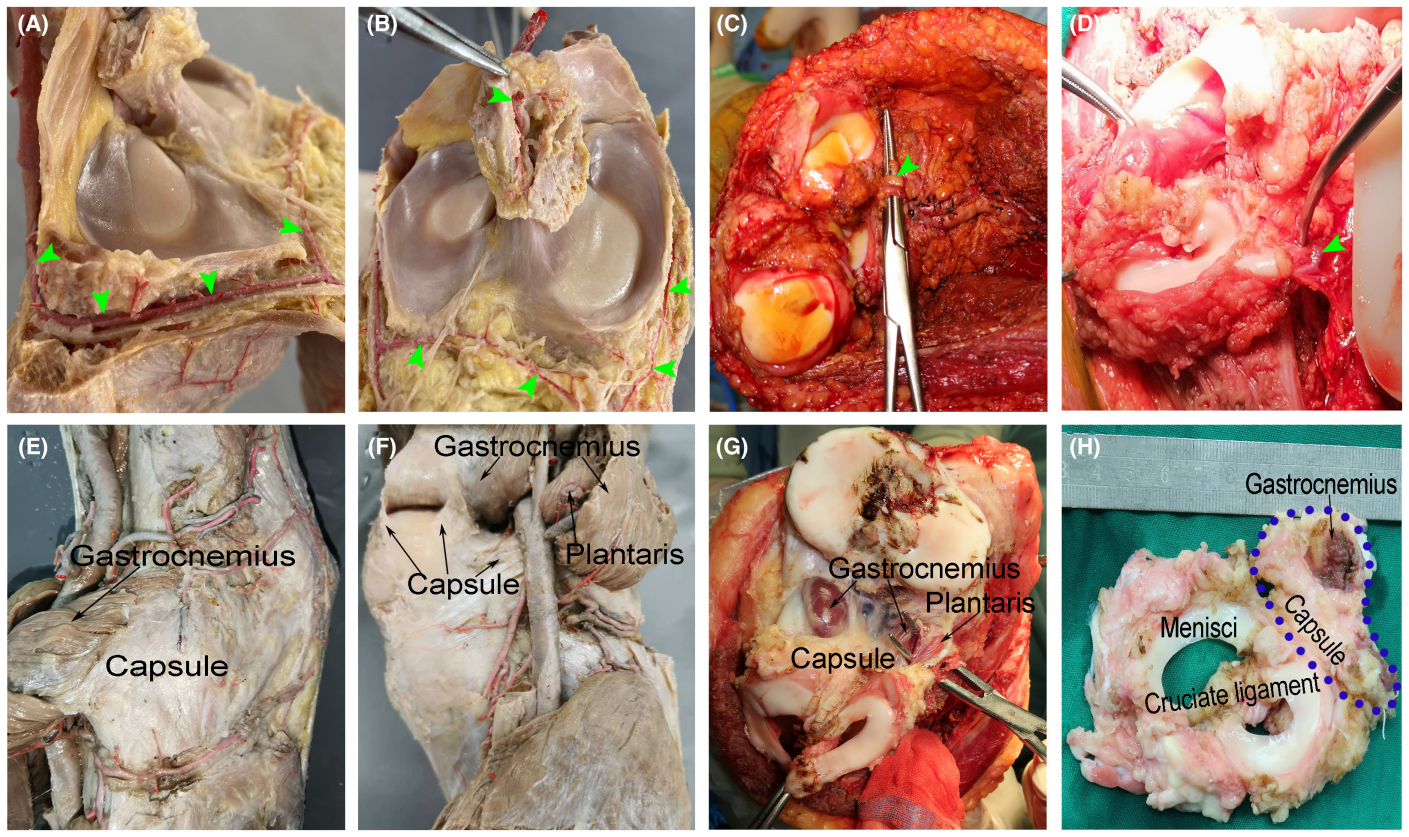
Implications for the design of composite. Vascularity of menisci and cruciate ligaments in specimens (A, B) and intraoperative photographs (C, D) (green triangle indicates blood supply to composite). The heads of the gastrocnemius and plantaris arise from subjacent areas of the posterior capsule (E, F). Leaving some attachment of the posterior capsule and the muscle to the composite (blue circle) provides more tissue for further reconstruction (G, H).

The vascularity of the menisci, cruciate ligaments, and surrounding tissues indicated that although the inner portion of the menisci was relatively devoid of blood supply, the menisci, and cruciate ligaments were rich in blood vessels from the infrapatellar vascular plexus anteriorly, vascular synovial tissue and middle geniculate artery posteriorly, and vessel plexus at the periphery (Figure 1A–D). Notably, the nutrient vessels arose posteriorly to anteriorly, which indicated that to retain these vessels, the composite should be dissected out anteriorly to posteriorly.

Additionally, both heads of the gastrocnemius and plantaris arise from subjacent areas of the posterior capsule and oblique popliteal ligament (Figure 1E–H). These results indicate that if the defect was too large to be covered with the composite alone, some attachment of the posterior capsule and muscle to the composite could be retained to provide more tissue for reconstruction.

### 3.2 Surgical technique

All patients underwent wide tumor resection according to oncologic principles.^1^ At the study cohort, after wide resection, the location of the soft tissue defect and the laxity of the vascular pedicle of the composite was evaluated by the surgeon. Based on the evaluation, free or vascularized composite reconstruction was chosen. In general, where the defect was too distant to be attached, the free composite was used; otherwise, the vascularized composite was applied. The operative pathological biopsy was performed to make sure that the recycled composite was tumor freed with a negative surgical margin.

**FIGURE 2:**
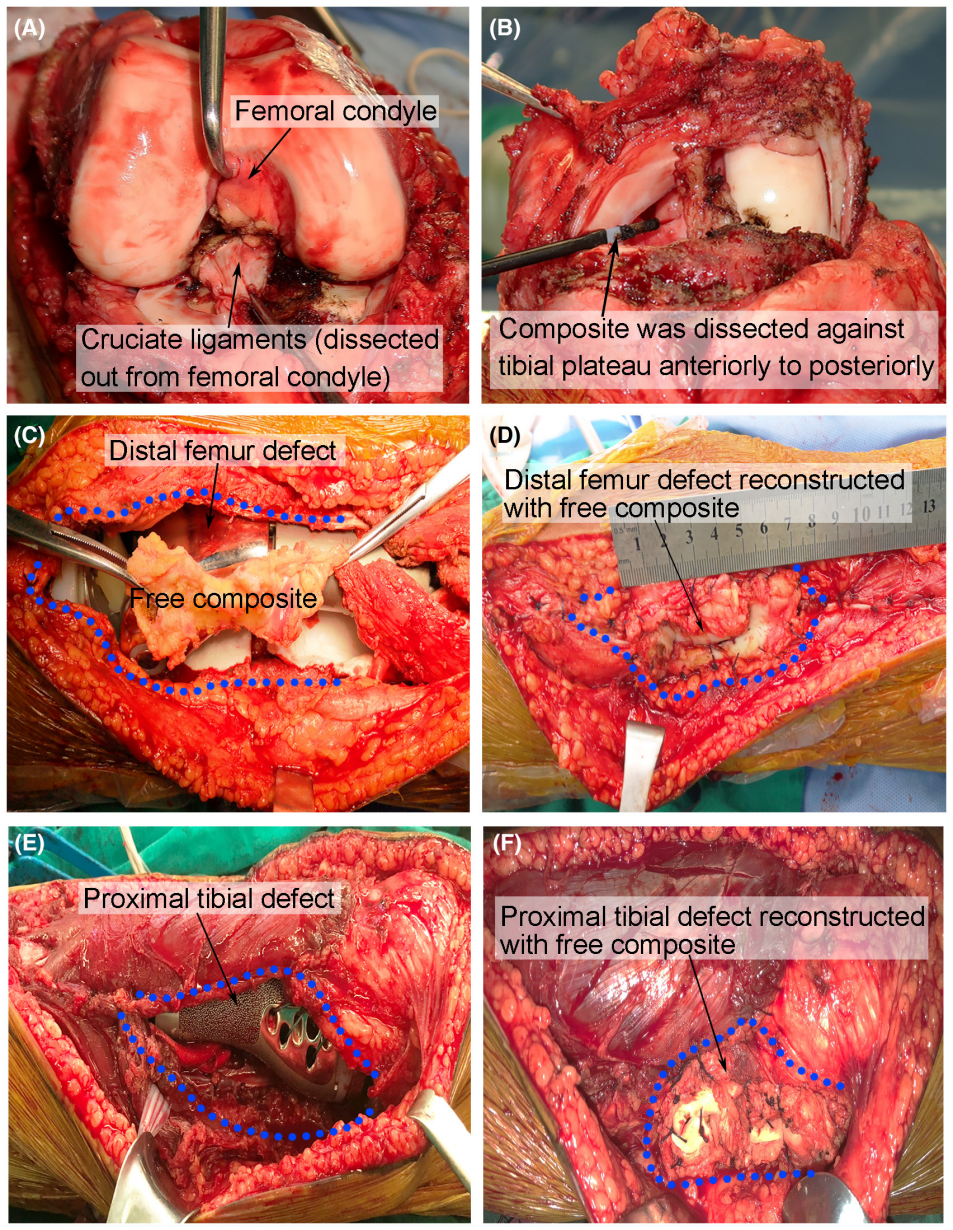
Reconstruction with free composite. Harvesting the free composite (A, B). First, the cruciate ligaments were dissected out from the femoral condyle (A), and then the composite was dissected out en bloc with a circumferential incision against the tibial plateau anteriorly to posteriorly (B). The composite was then used to cover the distal femur defect (C, D) or proximal tibial defect (E, F) and sutured directly to the residual muscles. The blue circle indicates the soft tissue defect covered with composite.

For the free composite, menisci and cruciate ligaments were dissected out en bloc with a circumferential incision around the tibial plateau anteriorly to posteriorly (Figure 2A,B). The size and extent of the defect were evaluated by the surgeons. If the defect was too large to be covered with the composite alone, some attachment of the posterior capsule and the plantaris and/or gastrocnemius to the composite was left to provide more tissue for subsequent reconstruction without compromising the safe distance. The composite was transferred to the defect and partially replaced the resected muscles. The free composite reconstruction was learned from the surgical technique of meniscal allograft^12^, ^13^ that the periphery of the composite was sutured to the residual muscles with bioabsorbable sutures and without anastomosing any vessels (Figure 2C–F).

**FIGURE 3:**
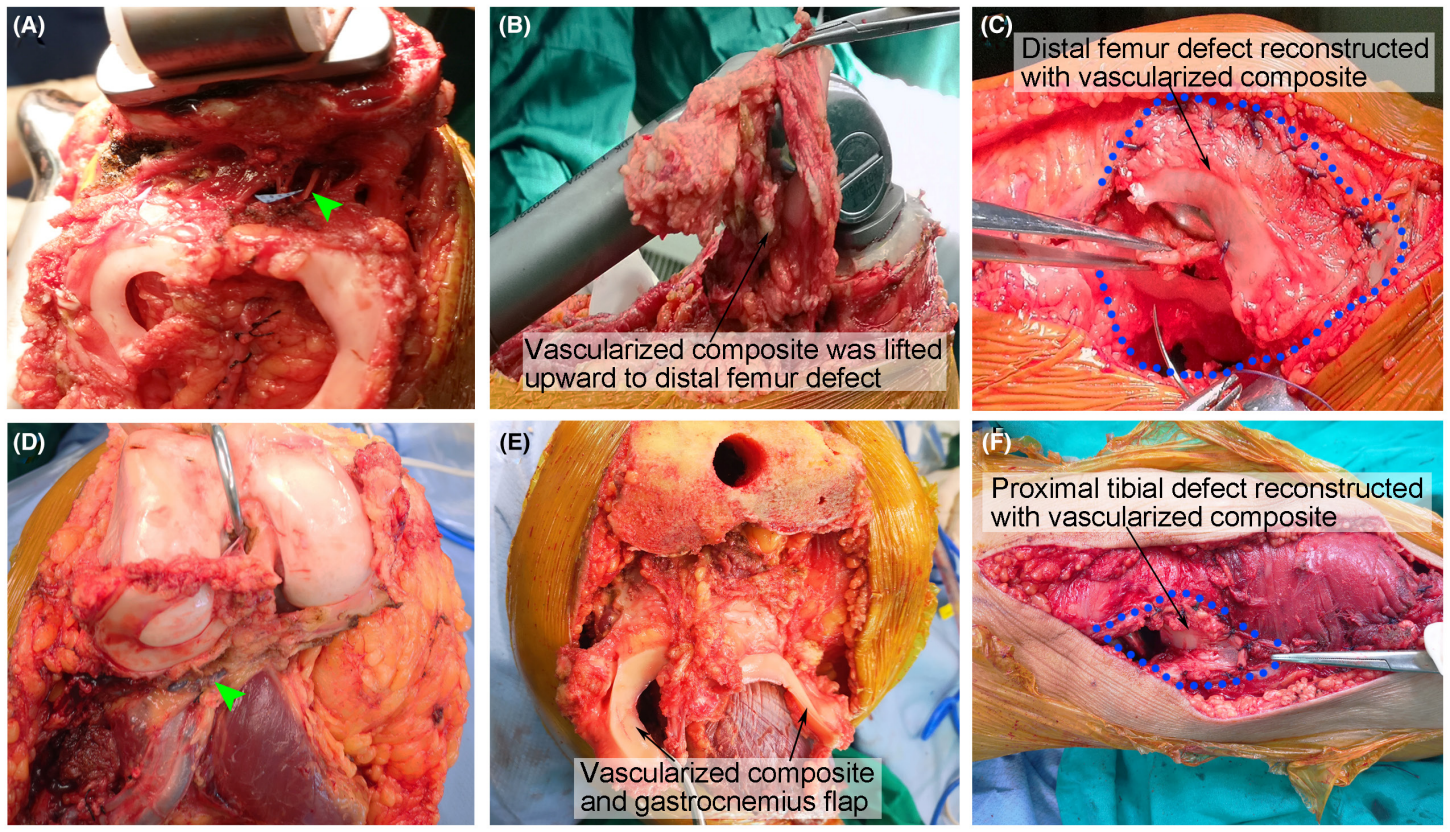
Reconstruction with vascularized composite. The vascularized composite was transferred to a distal femur defect (A–C) or proximal tibial defect (D–F). The green triangle indicates the blood supply to the composite. The blue circle indicates a soft tissue defect covered with composite.

The vascularized composite was circumferentially dissected anteriorly to posteriorly, and a perimeniscal tissue margin >0.5 cm containing peripheral vessels was left. The popliteal vessels and their branches entering the menisci and cruciate ligaments were isolated and preserved. For reconstructions in the femur, the composite was lifted upward and then rotated around the prosthesis to the soft tissue defects (Figure 3A–C). In all cases of tibial reconstruction, the vascularized composite was rotated to the anterolateral defect that remained after medial gastrocnemius flap transfer (Figure 3D–F). Careful attention was given to the laxity of the supplying vessels.

For the distal femur tumors, 10 vascularized and 19 free composites were sutured to the remaining muscles to cover the prosthesis and provide support for the reconstruction of the extensor mechanism (Figures 2C,D and 3A,C). For tibial reconstructions, the patellar tendon remnant was secured to the prosthesis first, and then the gastrocnemius flap was rotated around to cover the prosthesis. An anterolateral defect remained, so the composite was subsequently transferred to the defect (Figures 2E,F and 3E,F). The composite ensured both defect reconstruction and appropriate tension for patellar tendon reconstruction. To enhance the porosity of the composite, the femoral and tibial surfaces of the menisci were punched with a syringe needle (7G) to facilitate ingrowth from the surrounding tissues.^14^

Intraoperative photographs (Figures S1 and S2) and intraoperative videos (see Video, Online Supplementary Video) are presented to demonstrate the use of menisci–cruciate ligament composite.

### 3.3 Clinical and oncologic outcomes

Soft tissue reconstruction with autologous menisci–cruciate ligament composite was performed in 40 patients. Fourteen of these patients were treated with vascularized composite transfer (10 distal femurs and four proximal tibias), and 26 were treated with free composite transfer (19 distal femurs and seven proximal tibias). The composite can be used to cover soft tissue defect areas of 22 to 48.38 cm^2^ (34.67 ± 6.48 cm^2^). The follow‐up period was more than 12 months for each patient (16 patients >30 months, 14 patients >20 months, range, 13–39 months). No statistically significant differences were found in age, gender, resected tumor size, surgical approach, and pathology between the study and control cohorts (Table 1).

**TABLE 1:** Sociodemographic and clinical characteristics of patients in study and control cohorts

Characteristic	Study cohort (reconstructed with composite [*n* (%)])	Control cohort (reconstructed without composite [*n* (%)])	*p* value
Age at surgery (year)			0.494
≤18	32 (80%)	64 (74.4%)	
>18	8 (20%)	22 (25.6%)	
Gender			0.344
Male	25 (62.5%)	61 (70.9%)	
Female	15 (37.5%)	25 (29.1%)	
Location of tumor			0.410
Distal femur	29 (72.5%)	56 (65.1%)	
Proximal tibia	11 (27.5%)	30 (34.9%)	
Surgical approach			0.482
Anterior knee incision	1 (2.5%)	7 (8.1%)	
Anterior medial knee incision	32 (80%)	65 (75.6%)	
Anterior lateral knee incision	7 (17.5%)	14 (16.3%)	
Resected tumor size (cm^3^)^a^			0.678
≤250	10 (25.5%)	23 (26.7%)	
250–500	19 (47.5%)	34 (39.55)	
>500	11 (27.5%)	29 (33.7%)	
Pathology			0.952
Others	1 (2.5%)	2 (2.3%)	
Osteosarcoma	39 (97.5%)	84 (97.7%)	0.535
Conventional OS	38	83	
Telangiectatic OS	1	0	
Low‐grade central OS	0	1	

^a^ The resected tumor size was measured in the dimension of resected specimen and was calculated according to the formula for the volume of an ellipsoid mass = [(π*length*width*depth)/6].

**FIGURE 4:**
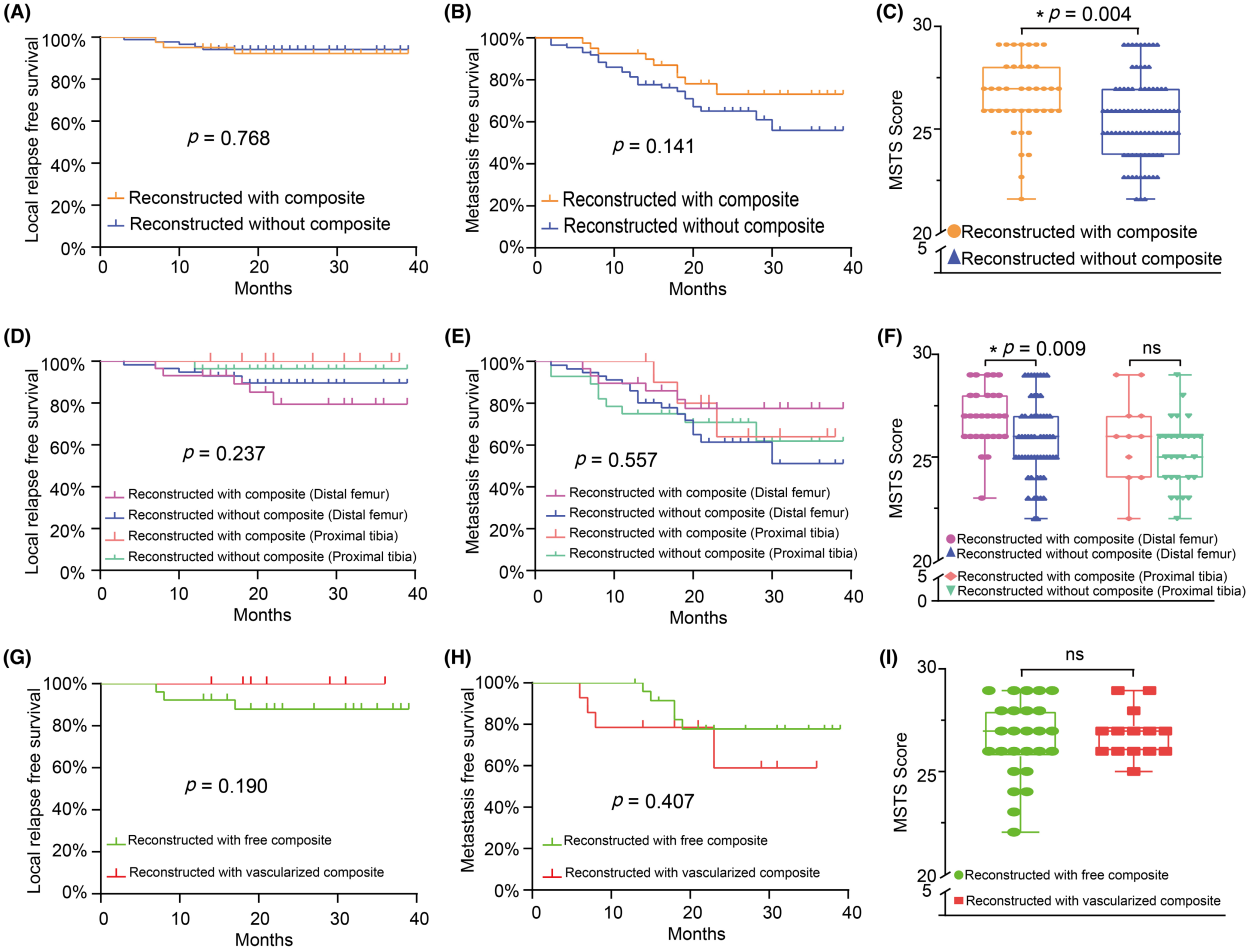
Oncologic and functional outcomes. Kaplan–Meier‐free survival analysis of 2‐year local relapse‐free survival (LRFS) (A) and 2‐year Metastasis‐free survival (MFS) (B) between the study and control cohort. Results of Musculoskeletal Tumor Society (MSTS) score between study and control cohort (C). Kaplan–Meier free survival analysis of 2‐year LRFS (D) and 2‐year MFS (E) between subdivisions. Results of MSTS score between subdivisions (F). Kaplan–Meier‐free survival analysis of 2‐year LRFS (G) and 2‐year MFS (H) between patients reconstructed with free and vascularized composite. Results of MSTS score between patients reconstructed with free and vascularized composite (I). (*p < 0.05, ns indicates not significant).

The oncologic outcomes of 2‐year local replase‐free survival (2‐year LRFS) were 92.21% and 94.13% (*p* = 0.768), and 2‐year metastasis‐free survival (2‐year MFS) were 73.24% and 65.12% (*p* = 0.141), respectively, without significant difference between the study and control cohorts (Figure 4A,B). Furthermore, there were no differences between the same subdivision with regards to 2‐year LRFS (*p* = 0.237) and 2‐year MFS (*p* = 0.557) (Figure 4D,E). No statically significant difference was noted in 2‐year LRFS (87.91% vs. 100%, *p* = 0.190) and 2‐year MFS (77.76% vs. 58.93%, *p* = 0.407) between patients reconstructed with free or vascularized composite (Figure 4G,H).

### 3.4 Functional outcomes

The overall mean MSTS score in the study cohort was 26.68 points (88.9%, range, 21–29). Low scores were seen in the patients with local recurrence and PJI. Apart from these patients, the mean MSTS score was 27.03 points (90%). The mean MSTS score differed between the study and control cohort (26.68 vs. 25.66, *p* = 0.004, not unpaired *t‐*test) (Figure 4C). Of note, MSTS score was higher for patients reconstructed with distal femur subdivision compared with the same subdivision in the control cohort (26.97 vs. 25.90, *p* = 0.009, not unpaired *t* test) (Figure 4F). Patients reconstructed with composite at proximal tibia had high MSTS scores compared with the same subdivision in the control cohort but this did not reach a statistically significant difference (25.91 vs. 25.18, *p* = 0.260, not unpaired *t‐*test) (Figure 4F). Patients reconstructed with free or vascularized composite had comparable MSTS scores (26.58 vs. 26.86, *p* = 0.622, not unpaired *t*‐test) (Figure 4I). These results indicated better functional capability for patients reconstructed with composite, especially for those reconstructed at the distal femur.

### 3.5 Complications

There was no perioperative complication in all patients in the study cohort. PJI occurred in three patients in the study cohort (3/40, 7.5%) at 7, 8, and 18 months postoperatively (Patients: S‐P06, S‐P07, and S‐P11, see Table S1). Notably, there were no postoperative complications for patients receiving vascularized autologous composites in the study cohort, though no significant difference compared with free composite subdivision (*p* = 0.186, chi‐square test). Ten patients in the control cohort (11.6%) developed complications, including PJI (*n* = 4) and wound complications (*n* = 6). There was no difference between the study and control cohort regarding complications (*p* = 0.478, chi‐square test).

### 3.6 Postoperative color doppler ultrasound and histopathologic analysis

**FIGURE 5:**
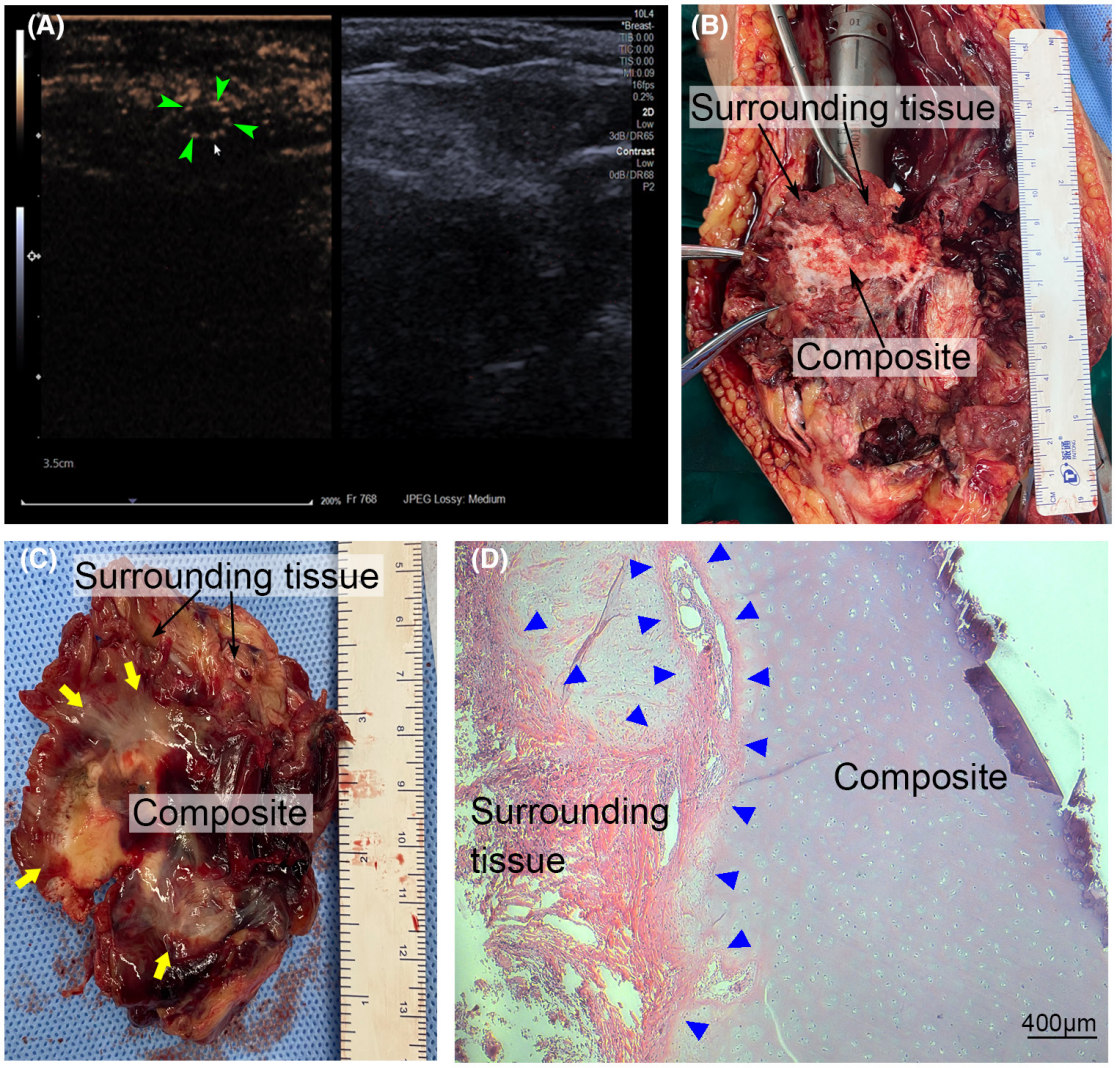
Postoperative color doppler ultrasound and analysis of a retrieved composite. Peripheral rim healing and dotted blood flow signals at the side of the anastomosis (green triangle) were detected with contract‐enhanced ultrasound 16 months postoperatively on a patient who was reconstructed with free composite (A). Gross macroscopic remodeling of a retrieved composite from an amputated limb 17 months postoperatively indicated healed well with surrounding tissues (B). On the articular surface side, the composite acted as a scaffold covered by aligned collagen fibers characteristic of synovium‐like membranes (yellow arrow), with a smooth transition from the surrounding tissues to the composite (C). Hematoxylin and eosin staining of the retrieved composite indicated the connection between the composite and surrounding tissues (blue triangle) (D).

Peripheral rim healing and dotted blood flow signals at the side of the anastomosis were detected with contract‐enhanced ultrasound 16 months after surgery (Figure 5A) in a patient (S‐P26, Table S1) who was reconstructed with free composite following femur tumor resection in the study cohort.

A composite was harvested from the amputated limb of S‐P25 (Table S1) in the study cohort. On gross examination, white organized tissue, mainly at the anastomoses, conferred actual healing with the surrounding tissues (Figure 5B). On the articular surface, the composite acted like a scaffold covered with aligned collagen fibers characteristic of synovium‐like membranes, with a smooth transition from the surrounding tissues to the composite (Figure 5C). Histological analysis showed living cells in the composite and clear integration of the composite with the surrounding tissues. (Figure 5D).

## 4 DISCUSSION

The goal of soft tissue reconstruction is to provide reliable and stable coverage.^15^ Despite significant improvements in oncological treatment, the management of soft tissue defects following endoprosthetic reconstruction remains challenging. In the knee region, muscle flaps, such as from the gastrocnemius or soleus muscle, are a standard treatment option.^16^

Due to the complexities of tumor size, tumor location, and the range of oncologic resection, these flaps are not sufficient to cover the exposed implant or vital structures in cases of large defects. As indicated in the study cohort, although we adopted an approach to augment the surface area of the gastrocnemius flap by selectively excising the muscle belly and removing the gastrocnemius fascia, allowing the muscle flap to unfurl, and an increase in coverage was achieved,^17^ a soft tissue defect still presented in the lateral superior region.

Additionally, defects in the distal femur are usually reconstructed with vasti, gracilis, semimembranosus, or sartorius.^4^ These muscles, in various proportions, are frequently involved in the tumor mass and therefore are removed together with the neoplasm. For the gastrocnemius muscle to be used in the reconstruction of distal femur defects, the muscle belly cannot be in the field of dissection during distal femoral tumor resection, so there is a need for further dissection. Moreover, the coverage capacity of gastrocnemius flaps in the treatment of distal femur defects is relatively limited.^18^, ^19^

### 4.1 Inspiration and theoretical basis for applying autologous menisci–cruciate ligament as a flap

The notion that applying autologous menisci and cruciate ligament as a supplementary flap for soft tissue coverage originated from a pioneering study in which a free flap was harvested from an amputated leg for distant reconstruction.^7^ Meniscal allograft has also shown favorable results in terms of reconstruction of the meniscus and yields high rates of survivorship in short‐, mid‐, and long‐term studies.^12^, ^20^ The autologous tendon (such as the patellar tendon, semitendinosus, or gracilis) has been widely used in anterior cruciate ligament reconstruction.^21^ Some histologic and histochemical studies have also confirmed the cell distribution and regenerative activity within these transplants.^22^

### 4.2 Effectiveness of autologous menisci–cruciate ligament composite in repairing soft tissue defects

The composite had the size and bulk necessary to eliminate the local wound tension and obliterate the dead space. In this study, the composite was used to cover soft tissue defect areas of 22–48.38 cm^2^ and sutured with the remaining muscles to cover the prosthesis and provide support for the reconstruction of the extensor mechanism. As indicated in patients in the study cohort, following distal femur tumor resection, without harvesting of a gastrocnemius muscle flap, meniscus–cruciate ligament composite acted as a supplemental flap and coupled with residual muscles, such as vasti, gracilis, semimembranosus, or sartorius, was able to provide adequate coverage. For reconstruction of the proximal tibial defect, the composite acted as a good supplement for the defect that remained after gastrocnemius flap rotation. With contract‐enhanced ultrasound, peripheral rim healing and dotted blood flow signals at the side of the anastomosis were detected in a patient who was reconstructed with free composite following femur tumor resection.

Although free composite may compromise the blood supply and undergo histologic translation,^23^ it can provide a customized shape and appropriate repair for most defects, especially distant defects. Furthermore, there was no significant difference in complications compared with vascularized composite. Gross macroscopic remodeling and histopathologic analysis of a retrieved free composite from one patient after 17 months of implantation indicated good healing with the surrounding tissues. Further investigation should focus on the process of histologic and biochemical changes in composites in vivo and develop methods to accelerate the remodeling of composites into anastomoses with the surrounding tissues.

Apart from providing soft tissue coverage, the composite, as the tendinous structure, was sutured with the residual muscles to provide support for the reconstruction of the extensor mechanism. In the study, the mean MSTS score was 26.68 points (89.9%, range, 21–29), and low scores were seen in the patients with local recurrence and PJI. Our study had similar functional results or higher MSTS scores compared with those reported in the literature.^24^, ^25^ Notably, the patients reconstructed with composite had higher MSTS scores compared to not only the control cohort but also an earlier report from our institute (26.68 vs. 25.5).^9^

### 4.3 Analysis of complications and events

In the more than 12‐month follow‐up period, the overall postoperative complication rate, including that of PJI, in our study was 7.5% (3/40), which was comparable to the control cohort and the reports in the literature.^26^, ^27^ We reviewed the records of our infected patients to ascertain the potential causes of infection. Two of them had severe adjuvant chemotherapy‐induced myelosuppression, and the other had undergone irregular puncture and drainage in a local hospital. Several well‐designed retrospective and prospective studies confirmed that chemotherapy‐induced myelosuppression and additional operation were risk factors for postoperative infection.^28^, ^29^ These factors were present in our infected patients and indicated that the composite was not the cause. The recurrent ratio in the study cohort was 7.5%, which was comparable to the control cohort and some literature reports as well. Recurrence as documented in the study cohort is most likely caused by the disease itself.

A limitation of this study was that only 126 patients with malignant tumors around the knee were involved due to the paucity of malignant bone tumors available. The in vivo remodeling of the free composite was investigated, but no vascularized one was observed. A long‐term follow‐up study is still underway, and more results on the clinical outcomes will be obtained over time.

## 5 CONCLUSION

In summary, the results of this study support the use of autogenous menisci–cruciate ligament composite for soft tissue coverage following endoprosthetic reconstruction around the knee. The composite provides an alternative or supplementary option for soft tissue reconstruction. Either vascularized or free composite can be applied, depending on the size and localization of the defect, without additional expense.

## AUTHOR CONTRIBUTIONS

**Yu Xu:** Data curation (lead); writing – original draft (lead); writing – review and editing (equal). **Yan Li:** Validation (equal); writing – original draft (equal). **Yiwei Fu:** Writing – original draft (equal); writing – review and editing (lead). **Bo Wang:** Visualization (equal). **Tiao Lin:** Investigation (equal). **Changye Zou:** Investigation (equal). **Gang Huang:** Formal analysis (equal). **Jingnan Shen:** Conceptualization (equal). **Junqiang Yin:** Conceptualization (lead); methodology (lead); writing – original draft (supporting).

## CONFLICT OF INTEREST

The authors have no conflict of interest relevant to this article.

## ETHICAL APPROVAL STATEMENT

This study was approved by the ethics committee of the First Affiliated Hospital of Sun Yat‐Sen University (Application ID: [2021]119) and registered at ChiCTR (ChiCTR2100052249).

## Supporting information


Figure S1.

Figure S2.

Table S1.

Table S2.

Video S1.

## Data Availability

The individual patient characteristics in the study and control cohorts are shown in Tables S1 and S2, respectively. Data from the intraoperative operations are provided in Supplementary Video and Figures S1 and S2.
