# Supplementary figures and images for: Autologous menisci–cruciate ligament composite as a flap for soft tissue reconstruction following malignant bone tumor resection around the knee
Source: Cancer Med. 2023 Jan 9;12(7):8027–37. doi: 10.1002/cam4.5591 (PMC10134327; doi:10.1002/cam4.5591)

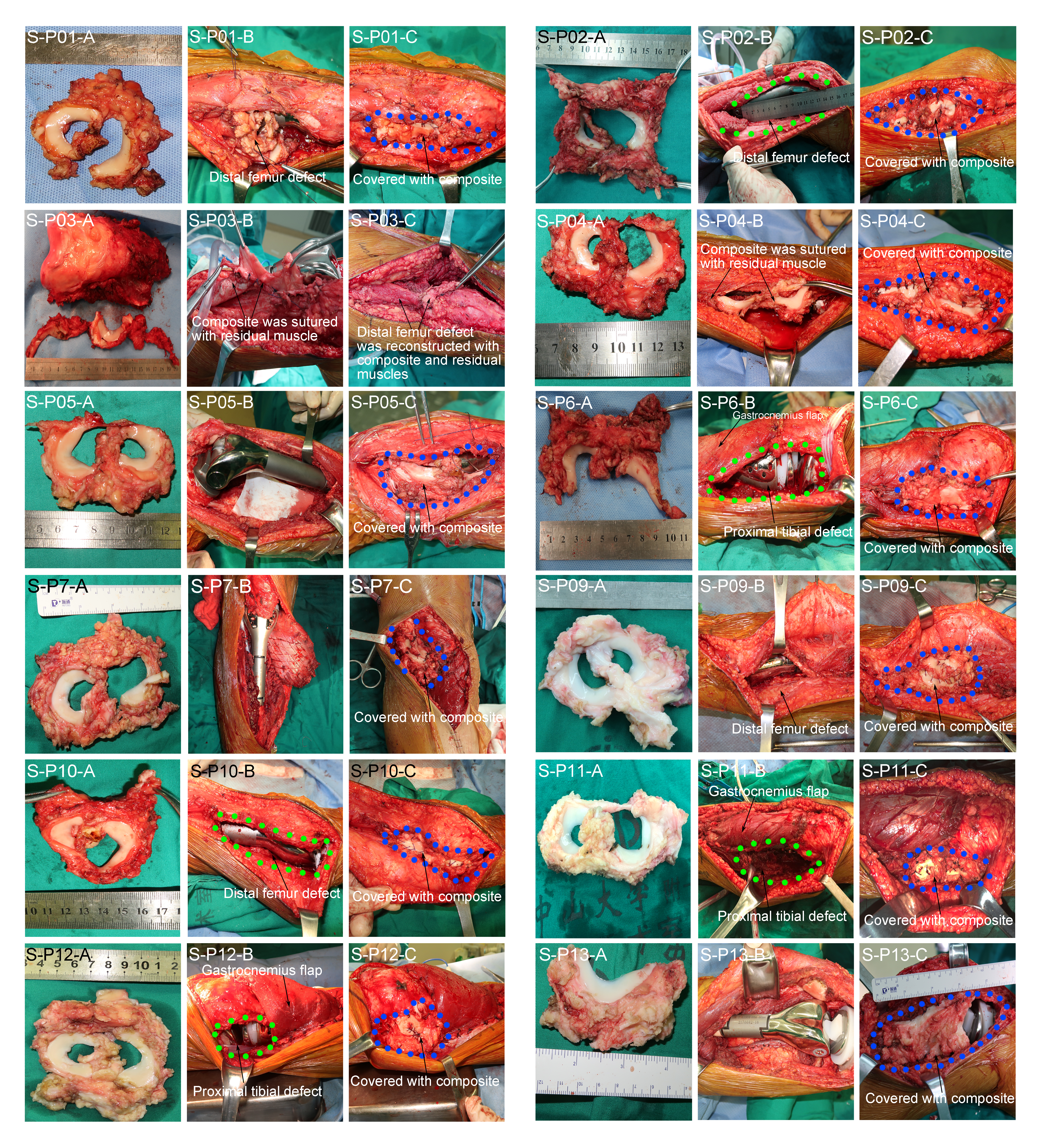

Supplement: Supplementary file 1 — Figure S1. [file CAM4-12-8027-s005.tif]

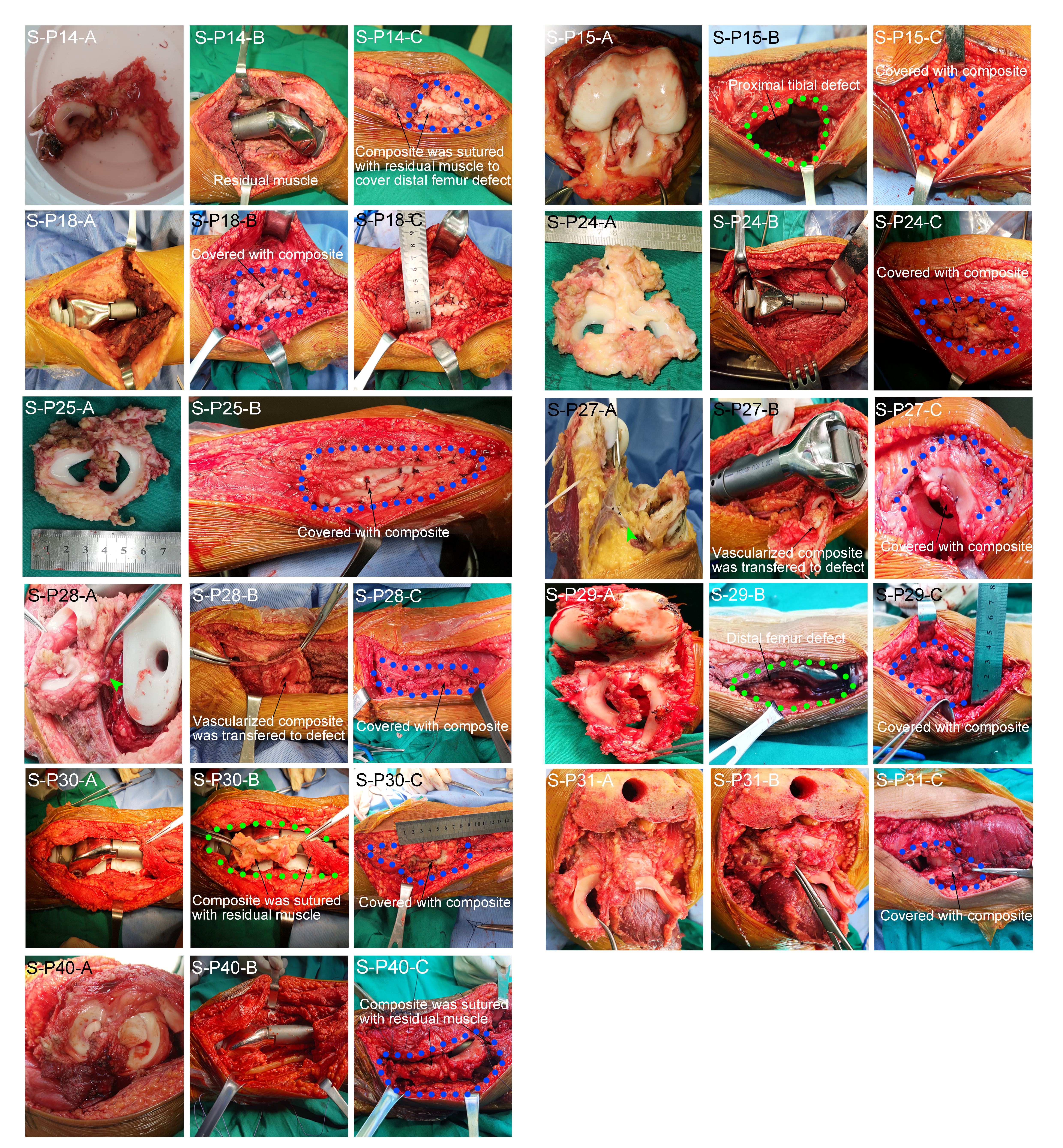

Supplement: Supplementary file 2 — Figure S2. [file CAM4-12-8027-s004.tif]
